# Recent Advances in Coordination–Insertion Copolymerization of Ethylene with Polar Monomers Catalyzed with Pd and Ni Complexes

**DOI:** 10.3390/polym18101243

**Published:** 2026-05-19

**Authors:** Suling Hu, Yi Zhou, Hongfan Hu, Guoliang Mao, Shixuan Xin

**Affiliations:** 1 Provincial Key Laboratory of Polyolefin New Materials, College of Chemistry & Chemical Engineering, The Northeast Petroleum University, Daqing 163318, China; 18276404935@163.com (S.H.); maoguoliang@nepu.edu.cn (G.M.); 2 Petrochina Petrochemical Research Institute, Petrochina Company Limited, Beijing 102206, China; zhouyi9@petrochina.com.cn (Y.Z.); huhongfan@petrochina.com.cn (H.H.)

**Keywords:** functionalized polyethylene, polar monomer, coordination–insertion polymerization, catalysis, Ni, Pd

## Abstract

The incorporation of polar functional groups into polyethylene (PE) chains at controlled concentrations enables tailored multi-functionality, manifesting as printability enhancement, improved dyeability, and enhanced blending compatibility with diverse polymeric materials. The most effective way to incorporate polar monomers into the PE macromolecules is the transition metal-mediated coordination–insertion copolymerization of ethylene with polar monomers. However, the Lewis basic heteroatoms (N, O, S, P, etc.) in polar monomers are prone to strongly coordinate to the catalytic center, resulting in irreversible catalyst deactivation. Owing to the nature of tolerance to Lewis basic functionalities, rationally designed Pd and Ni complexes have proven to catalyze direct coordination polymerization of ethylene with polar monomers, which opened a practical way to prepare functionalized polyethylenes (F-PEs). In this context, we summarize the recent advances of the Pd and Ni complexes catalyzed copolymerization of ethylene with various polar monomers, especially focused on those commercial polar monomer feedstocks. In addition, the effects of metal, ligand structural modification, and additives regulation on the catalytic performances were analyzed in detail. Some key ideas on the salient aspects of the catalyst are presented, and the challenges and prospects of Pd and Ni catalysts in the polar monomer copolymerization problems are also discussed.

## 1. Introduction

Polyolefins (POs) are widely used commodity synthetic resins that are cost-effective and offer excellent comprehensive performance in modern daily life. Polyethylene (PE) is one of the most extensively applied POs due to its indispensable mechanical properties, easy processing, chemical stability, and electrical insulation properties [1,2,3,4], and PEs are produced at over a hundred million metric tons per annum worldwide. However, PE is a non-polar polymer with a saturated hydrocarbon backbone, which makes it incompatible with the majority of polar materials, thus severely limiting its application scenarios.

In order to overcome the shortcomings of PE applications, polar functionalization became a research hot spot [5,6,7,8]. Polar functionalization of PE can be divided into three technical categories: (1) olefin metathesis polymerization, (2) post-functionalization, and (3) metal-catalyzed coordination–insertion copolymerization.

Olefin metathesis polymerization, encompassing both ring-opening metathesis polymerization (ROMP) of cyclic olefins and acyclic diene metathesis (ADMET) polymerization, requires precise synthesis of specific cyclic monomers. However, the intricate and costly synthetic procedures for cyclic comonomer preparation, coupled with the requirement for post-polymerization catalytic hydrogenation, collectively hinder large-scale industrial implementation [9,10,11,12,13,14].

Post-functionalization is also called the radical grafting process, which grafts polar or reactive groups to the backbone of PE via a free radical mechanism. This process involves complex and energy-intensive reaction steps and is inevitably accompanied by intrinsic side reactions (for instance, crosslinking and degradation).

In contrast, the method of coordination–insertion copolymerization has more prominent advantages in the precise regulation of copolymers and simple polymerization reaction steps [15,16,17,18]. There is great potential to achieve the commercialization of functionalized PE (F-PE) through this method. Therefore, the coordination–insertion copolymerization of ethylene (E) with polar monomer is still the major focus of research on F-PE.

There are three key elements governing the coordination–insertion polymerization: the transition metal type, the ligand structural environment, and the nature of the polar monomer. Due to the strong Lewis acidic nature of the early-transition metals (such as IIIB and IVB group metals), their catalytically active species readily form stable chelates with polar monomers, causing the catalysts’ irreversible deactivation. In comparison, the less Lewis acidic late-transition metal complexes can tolerate the polar group poisoning effect under favorable reaction conditions [19,20,21,22], and inevitably, the rationally designed late-transition metal complexes become the spotlight of the catalytic copolymerization of ethylene with polar monomers.

Brookhart et al. [23] first discovered that the α-diimine Pd(II) catalysts catalyze direct copolymerization of ethylene (E) with acrylates, generating highly branched E-acrylate copolymers with the acrylate located basically all on the branch ends. Subsequently, Grubbs’ single-component Ni(II) catalysts [24] enabled the copolymerization of ethylene with ester-substituted norbornene (NB), to form linear copolymers with high incorporation of the 4-ester-substituted NB. Since then, late-transition metal catalysts have been modified by subtle and more drastic changes to the ligand structure, which affects catalytic performance, thermal stability, copolymer structure, polar group insertion rate, etc. To date, many excellent reviews have summarized the regulation of copolymer structure by polar functionalities and catalyst heterogenization strategies [25,26,27,28,29]. These reviews provided comprehensive information regarding their potential in industrial utilization and facilitated their application in preparing functionalized PEs, gradually bridging the gap between late-transition metal catalysts preparation methods and practical application in E-polar monomer copolymerization. Moreover, considerable achievements have been made in the development of various new Pd and Ni complexes for catalytic copolymerization of E-polar monomers. Therefore, the “structure-activity relationship” of late-transition metal is further sorted out to guide the direction of future research.

In this context, we provide an up-to-date survey on the Pd and Ni catalysts for the copolymerization of E-polar monomers developed within the last several years. The relationship between ligand structure and thermal stability, copolymer molecular weight (MW), ratio of polar group insertion, and range of available polar monomers is discussed, and primary outlooks will be summarized toward the future development of Pd and Ni catalysts in the copolymerization of E-polar monomers.

For the convenience and clarity of the readers, the names of the polar monomers and their abbreviations used in this context are listed in Table 1, and the full name of each polar monomer is provided where it first appeared.

## 2. Pd Catalysts

The coordination–insertion copolymerization steps of E-polar monomer include the typical chain initiation, chain propagation, chain transfer/termination, and on frequent occasions, the chain walking. Chain walking is to recognize preferred bond-forming sites by allowing the movement of the catalytic center along the alkyl chain, thereby functionalizing “unreactive” C-H bonds to randomly form short alkyl branches along the PE backbone [30]. Initially, Brookhart et al. first reported that E-acrylates could produce copolymer under the influence of β-diimine Pd(II) catalysts [23,31,32]. Due to the symbolic chain-walking nature of the Pd catalysts, highly branched E-acrylate copolymer products were obtained with the polar groups almost exclusively situated at the branch ends. Subsequently, Dai et al. synthesized highly active Pd cationic catalysts, which effectively inhibited the chain-walking characteristic of the Pd catalyst through the spatial effect of the ligand structure [33,34,35,36,37,38,39]. High-molecular-weight and low-branch-density PE copolymers can be created, with most of the methyl acrylate (MA) located in the main chain. In recent years, new breakthroughs through modification of the ligands on the Pd complexes to regulate the microstructure and property of the copolymers have also been successful.

### 2.1. [N,N]-Type Ligands

**Scheme 1:**
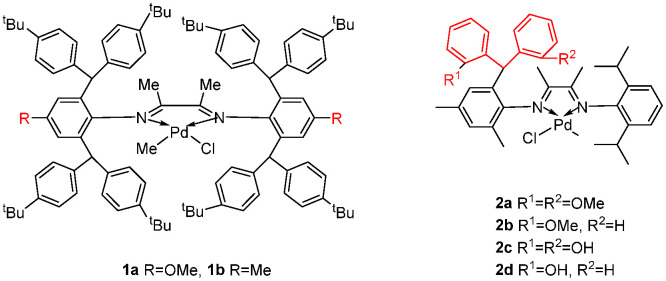
[N,N]-chelating Pd catalysts **1** and **2** regulated by steric hindrance and electronic effects.

Owing to the impressive catalytic capability in E-MA copolymerization and marked chain-walking mechanism in producing high-MW F-PE with highly branched structures, [N,N]-chelating Pd catalysts enjoyed long-term research interests in the last thirty years. On the other hand, concerning the low catalytic activity, insufficient mechanical properties of copolymers (e.g., strength and heat resistance) caused by excessive branching, and the suboptimal monomer incorporation ratios have severely hindered their practical applications. In 2020, Dai et al. [40] systematically explored the electron-donating effects of *para*-substitution on the N-aryl rings in α-diimine Pd complexes **1a, 1b** for copolymerization of ethylene with undec-10-enoic acid (E-UA) and its derivative methyl undec-10-enoate (E-MU, Scheme 1). Under identical reaction conditions (0.6 MPa E pressure, 30 °C), catalysts **1a** and **1b** exhibited moderate copolymerization activity and similar monomer incorporation ratios, yielding copolymers with high branching densities (61–76/1000 C). However, the methyl-substituted catalyst **1b** generated copolymers with higher MW (M_n_ up to 3.4 × 10^5^ g/mol) than **1a**, and the resulting material could serve as a polar-F-PE thermoplastic elastomer with enhanced elasticity. Broader catalyst screening within this study established that a branching density around 60/1000 C combined with high MW is critical for elastomeric performance; both lower and higher branching densities failed to achieve comparable elastic recovery. Within this *para*-substituted series, **1b** is thus the most effective catalyst for high-MW elastomers (Table 2).

Subsequently, Muhammad et al. [41] also studied the catalytic behavior of Pd complexes **2a–d** bearing *ortho*-methoxyl/hydroxyl-functionalized dibenzhydryl substituents for E-MA copolymerization (Scheme 1). **2a** displayed the highest activity (1.4 × 10^5^ g/(mol·h)) and produced high-MW PE (6.9 × 10^5^ g/mol). Notably, **2c** and **2d** bearing hydroxyl groups resulted in slightly higher incorporation of MA compared with **2a** and **2b** (Table 2). The authors suggested that fewer steric hydroxyl groups favor the incorporation of the steric-demanding MA. These two strategies highlight a clear trade-off in *α*-diimine Pd systems: *para*-methyl substitution (Dai) enhances MW and elastomeric properties, whereas *ortho*-hydroxyl functionalization (Muhammad) improves polar monomer uptake. Balancing these two objectives remains a key challenge for practical applications.

**Scheme 2:**
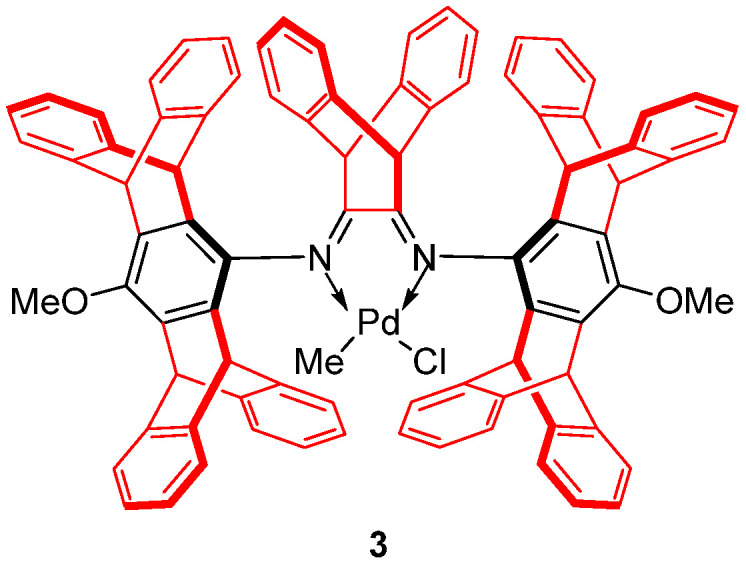
Pd catalyst **3** bearing rigid steric substituents.

Electronic effects do not always explicitly improve the catalytic properties of α-diimine Pd complexes. Zhang et al. [42] demonstrated this by employing a rigid steric modified Pd catalyst **3** (Scheme 2), where the contradiction between high branching density and in-chain polar monomer incorporation was explored. In E-MA copolymerization, **3** showed moderate catalytic activity (1.48 × 10^4^ g/(mol·h)) with notably improved thermal stability (operable from 30 °C to 90 °C, Table 2), producing copolymers with moderate MW (M_n_ up to 8.5 × 10^4^ g/mol), ultra-high branching densities (158/1000 C), and low MA incorporation (1.5 mol%). The rigid ligand framework limited the approach of acrylate to the Pd center and destabilized the more spatially demanding transition states, in contrast to catalysts with freely rotating dibenzhydryl substituents. Unlike the moderate branched elastomers obtained from Dai and Muhammad, **3** represents the most thermally robust α-diimine Pd system for E-MA copolymerization, though the hyperbranched structure and low polar monomer incorporation currently limit its applicability in high-strength or high-functionality materials.

The unusual regioselectivity observed with rigid catalyst **3** prompted further mechanistic consideration. Previous studies established that 2,1-insertion of MA into Pd-carbon bonds leads to partial in-chain incorporation, whereas 1,2-insertion typically forms a stable chelate through intramolecular coordination of the ester group to the metal center, which serves as the catalyst resting state [23,32,43,44,45,46]. In contrast, complex **3** favored 1,2-regioselective insertion of acrylate yet still resulted in in-chain MA incorporation, a behavior distinct from other *α*-diimine Pd complexes. Zhang et al. [47] further showed that **3** copolymerizes a variety of polar vinyl monomers, yielding ultra-high branched F-PE (branching density up to 207/1000 C) with functional groups incorporated both in-chain and at chain ends. The insertion mode proved highly dependent on the chelating ability and steric bulk of the polar monomer: strongly chelating groups such as amides or phosphonates suppressed ethylene coordination, while steric-demanding monomers like MMA exhibited slower insertion rates, making MA the most compatible comonomer among the polar analogues tested.

In a subsequent study, Zhang et al. [48] found that introducing polar additives during polymerization with **3** could dramatically alter copolymer topology. When acrylonitrile was used as the additive, the copolymer microstructure shifted from ultra-high branching density (158/1000 C) to medium branching density (49/1000 C), producing a polar functionalized diblock PE architecture difficult to access by other methods. Thus, the rigid framework of **3** offers dual advantages: exceptional thermal stability and additive-controlled microstructure regulation, complementing the elastomeric materials accessible from more flexible *α*-diimine systems.

**Scheme 3:**
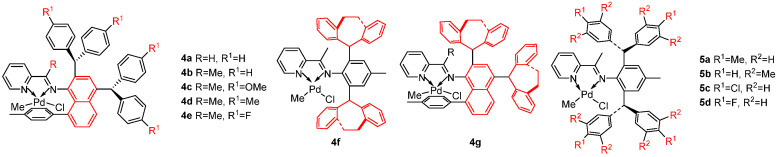
Iminopyridyl Pd catalysts **4**–**5** regulated by steric hindrance or electronic effects.

Expanding beyond *α*-diimine frameworks to target higher polar monomer incorporation, Li et al. explored iminopyridyl Pd catalysts. Complexes **4a**–**g** bearing dibenzosuberyl and/or 8-aryl naphthyl substituents (Scheme 3) produced hyperbranched PE with narrow PDI (<2) and MA incorporation up to 23% [49]. The steric properties of the imino N substituents governed both polar monomer incorporation and branching density, with **4a–e** and **4g** (dibenzosuberyl/8-aryl naphthyl) showing substantially higher MA incorporation than the *ortho*-dibenzosuberyl analog **4f**. A subsequent series **5a–d** bearing diarylmethyl substituents with remote electron-withdrawing or donating groups (Scheme 3) confirmed that electronic effects continue to influence performance. The *para*-Me-substituted **5b** improved MW and reduced branching density, though the M_n_ values remained low (6.3 × 10^2^ g/mol) with MA incorporation up to 15% [50] (Table 2). Overall, the iminopyridyl systems achieve significantly higher MA incorporation (up to 23%) than the *α*-diimine catalysts, positioning them as the preferred choice when maximizing polar functionality is the primary goal. Among these, **5b** offers the best balance of MW and branching control, though further gains in polymer chain length are needed for applications requiring mechanical integrity.

**Scheme 4:**
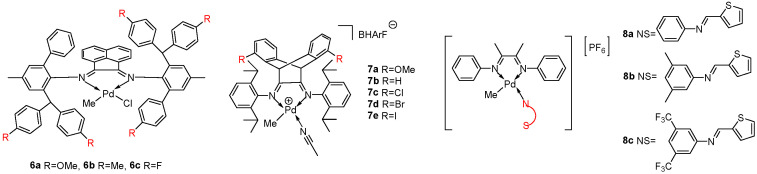
α-diimine Pd catalysts **6–8** with electron-effect substituents or ancillary ligands.

Further highlighting the beneficial role of electron-donating groups, Lu et al. [51] reported α-diimine Pd complexes **6a–c** with *ortho*-phenyl and *ortho*-diarylmethyl N-aryl substituents for E-MA copolymerization (Scheme 4). Complexes **6a** and **6b** bearing -OMe groups exhibited higher activity, MA incorporation (up to 10.7 mol%), and lower branching density than the -F-substituted **6c**, albeit overall activity remained low (~10^3^ g/(mol·h)) with moderate MW (~10^4^ g/mol) (Table 2). Similarly, Zheng et al. [52] demonstrated that electron-donating substituents on the ligand backbone of complexes **7** enhance catalytic performance, with the copolymer MW (M_n_ 2.94–3.84 × 10^4^ g/mol) and activity decreasing in the order OMe > H > Cl > Br > I (Scheme 4). These results consistently confirm that electronic enrichment, whether on the N-aryl rings or the backbone, improves performance in α-diimine Pd systems; nevertheless, the low activities and modest MW relative to the more optimized catalysts discussed earlier limit their practical relevance.

Whereas the previous examples focused on ligand electronics and steric effects, the role of external parameters, such as solvent, has also been explored to modulate copolymer microstructure. Drawing on the concept that coordinating solvents can serve as ancillary ligands to tune the reactivity of α-diimine Pd catalysts [53], Alberoni et al. [54] demonstrated the effect of solvents (dichloromethane and trifluoroethanol) and the α-diimine Pd complexes **8a**–**c** bearing N−S ligand for E-MA copolymerization. Dichloromethane is replaced with trifluoroethanol, and MA is inserted both in the backbone and at the branch ends in a ratio range from 9:91 to 45:55. The weak interaction between Pd ion and the sulfur atom was also found to be capable of facilitating the insertion of polar monomers into the polymer main chain.

**Scheme 5:**
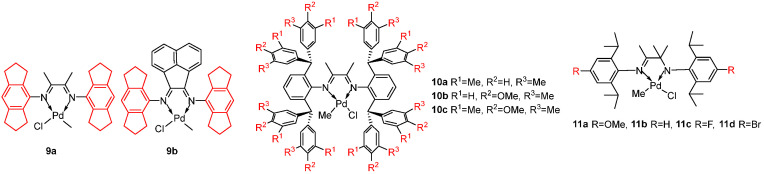
Pd catalysts **9**–**11** regulated by steric hindrance or electronic effects.

Steric openness at the Pd center continued to be a central theme in catalyst optimization. Wang et al. [55] designed α-diimine Pd complexes **9a** and **9b** bearing steric bulk bis(benzocyclopentyl) substituents (Scheme 5), which provide a more open axial space that facilitates MA insertion, achieving MA incorporation up to 20.7 mol%. Wu et al. [56] combined the steric effect of diarylmethyl groups with methoxy electronic effects in complexes **10a–c**, producing lightly branched, end-of-branch functionalized PE from ethylene copolymerization with MA, MU, or UA (Scheme 5). Complementing these steric approaches, a systematic electronic study by Zheng et al. [57] on complexes **11a–d** demonstrated that electron-donating groups promote E-MA copolymerization performance relative to electron-withdrawing substituents (Scheme 5); the latter increase electrophilicity and oxophilicity at the Pd center, strengthening chelation of polar intermediates and exacerbating catalyst poisoning. Consistent with this picture, subsequent studies have shown that weak OMe–Pd interactions not only enhance polar monomer copolymerization but also preserve chain-walking behavior, yielding branched copolymers [58,59]. Collectively, these studies reinforce that both open steric environments and electronic enrichment at the metal center converge to improve polar monomer incorporation while maintaining productive chain walking.

**Scheme 6:**
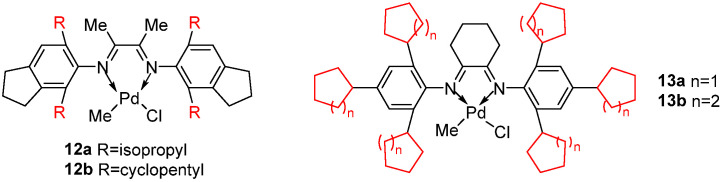
Pd catalysts **12**–**13** regulated by axial steric hindrance.

We continued with 60 developed catalysts **12a–b** bearing flexible axial steric substituents (Scheme 6), which produced branched copolymers with moderate MW (up to 1.37 × 10^4^ g/mol) and low MA incorporation (0.6–2.9 mol%) [60]. In contrast, Dai et al. [61] created **13a–b** (Scheme 6) bearing flexible cycloalkyl-substituted axial steric groups (Scheme 6), yielding branched copolymers with moderate MW (up to 4 × 10^4^ g/mol) and enhanced MA incorporation ranging from 1.53 to 4.54 mol%. The comparison between **12a–b** and **13a–b** suggests that cycloalkyl-based axial substituents offer a better balance of flexibility and steric protection than simple flexible chains, leading to simultaneous improvements in both MW and polar monomer uptake.

In general, these studies have shown that α-diimine-type [N,N]-Pd catalysts exhibit remarkable performance in E-MA copolymerization, enabling the precise synthesis of ultra-high branched main-chain ester-functionalized F-PE. Axial *ortho*-substituents with large steric hindrance on the N-aryl ligand represent a promising strategy that continues to be actively investigated. However, the development of catalytic systems that simultaneously achieve high catalytic efficiency, desirable polar monomer incorporation (>10 mol%), and high-MW copolymers remains a challenging goal.

**Table 2:** Ethylene-polar monomers copolymerization using [N,N]-Pd catalysts.

Entry	Cat.	Comon.	t (°C)	Act. *^a^*	X_m_ *^b^* (%)	M_n_ *^c^*	PDI *^c^*	B *^d^*	T_m_ *^e^* (°C)	Ref.
1	**1** **a**	MU	30	1.48	1.3	25.40	1.52	61	20	[40]
2	**1** **b**	MU	30	0.98	1.4	34.09	1.38	64	14	[40]
3	**2** **a**	MA	35	14	1.85	6.90	2.11	83	-	[41]
4	**2** **b**	MA	35	1.1	2.54	6.05	1.97	76	-	[41]
5	**2** **c**	MA	35	1.6	2.01	7.51	1.82	85	-	[41]
6	**2** **d**	MA	35	1.3	3.02	1.82	2.55	82	-	[41]
7	**3**	MA	90	1.48	1.5	1.48	1.48	158	-	[42]
8	**4** **c**	MA	40	0.065	22.7	0.56	1.18	134	-	[49]
9	**5** **b**	MA	40	0.13	15.05	0.63	-	117	-	[50]
10	**6** **a**	MA	40	0.14	10.7	0.31	1.40	118	-	[51]
11	**6** **b**	MA	40	0.15	8.5	0.33	1.20	113	-	[51]
12	**6** **c**	MA	40	0.1	7.6	0.24	1.30	121	-	[51]
13	**7** **a**	MA	25	0.37	2.07	3.84	1.06	103	-	[52]
14	**12b**	MA	30	1.37	0.8	2.62	1.68	86	-	[60]
15	**13b**	MA	30	0.33	1.53	4.00	1.40	97	-	[61]

*^a^* Activity = 10^4^ g/(mol·h). *^b^* X_m_ = comonomer incorporation (mol %). *^c^* determined by GPC, ×10^4^ g/mol, PDI as polydispersity index. *^d^* B = branching density: branches/1000 C, the terminal functional groups are added to the total branches. *^e^* Determined by differential scanning calorimetry (DSC).

### 2.2. [P,O]-Type Ligands

**Scheme 7:**
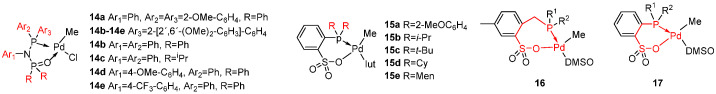
[P,O]-type Pd catalysts **14–17**.

Compared with *ɑ*-diimine [N,N]-type ligands, [P,O]-type chelating ligands offer distinct opportunities for producing characteristic polar F-PEs, due to the electronic asymmetry feature formed by the strong σ-donating phosphine and the σ-bonding oxide moieties, which drastically enhances the catalytic versatility of Pd catalysts and broadens the polar monomer scope. In a representative study, Chen et al. [39] developed a class of Pd complexes **14a–e** bearing diphosphazane monoxide-ligated structure and applied them in the ethylene copolymerization (Scheme 7). The catalytic performance of [P,O]Pd complexes **14a**–**e** was found to correlate with the short-bite ligand (increased ligand rigidity) and the electronic asymmetry of the ligand, as evidenced by NMR spectroscopy. In E-MA copolymerization experiments, **14a**–**e** exhibited moderate catalytic activity (10^4^ g/(mol·h)) and produced polymers with relatively low MW (10^3^ g/mol) and moderate incorporation of MA (up to 6.8 mol%). Furthermore, these [P,O]Pd complexes proved capable of direct E-acrylic acid (E-AA), E-butyl vinyl ether (E-BVE), and E-methyl methacrylate (E-MMA) copolymerization, though accompanied by a decrease in catalytic activity and polar monomer incorporation ratio.

[P,O]-type Pd catalysts have also enabled access to unconventional copolymer architectures through multi-component polymerization and chelate ring-size modulation. Wang et al. [62] studied the copolymerization of ethylene, carbene, and polar monomers by using Pd complex **15** catalyzes the three-component copolymerization of ethylene, carbene, and polar monomers to prepare an unprecedented F-PE with selective chain-end functionalization (Scheme 7). The resulting copolymers possessed low incorporation (0.1–1.45 mol%), low MW (M_n_~10^3^ g/mol), and selective distribution of the polar monomers in the main chain with carbene at the chain end, which together significantly influenced the mechanical and surface wetting properties. In a complementary study on chelating ring-size effects, Zou et al. [63] compared phosphine sulfonate Pd complexes **16** and **17**, which form seven- and six-membered chelating rings, respectively. Compared with the classic six-membered ring complex **17**, reduced catalytic performance was observed with **16**, attributed to the larger chelating ring increasing the flexibility of the Pd coordination sphere and reducing steric shielding toward the metal center (Scheme 7, Table 3).

**Scheme 8:**
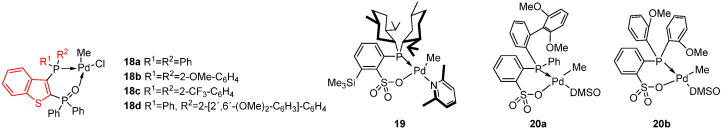
[P,O]-type Pd catalysts **18**–**20**.

Further improvements in thermal stability and activity were pursued through ligand rigidification. Mu et al. [64] reported few Pd complexes **18a**–**d** bearing diarylphosphine diphenylphosphinoxide (Ar_2_P-Ph_2_PO) ligands based on a benzothiophene backbone (Scheme 8). These complexes exhibited good thermal stability and high catalytic activity (10^6^ g/(mol·h)). Notably, **18b** displayed living behavior in ethylene copolymerization with a wide range of polar monomers, including MA, AA, ethenyltrimethoxysilane (VTMoS), allyl acetate (AAc), vinyl acetate (VAc), acrylonitrile (AN), and 6-chloro-1-hexene (6-Cl-Hex). Improved thermal stability was also a target for Tsuge et al. [65], whose complex **19** (Scheme 8) was applied to the copolymerization of ethylene with *tert*-butyl 5-norbornene-2-carboxylate (*t*BuNBE). Extending this approach to norbornene-based comonomers, Xu et al. [66] employed Pd complex **20** (Scheme 8) for the copolymerization of ethylene with disubstituted norbornene-based comonomers (*endo*-NB-ole, *endo*-NB-cin, *exo*-NB-ole, and *exo*-NB-cin), achieving remarkably high catalytic activity up to 2.04 × 10^6^ g/(mol·h), high MW (1.88 × 10^5^ g/mol), and high monomer incorporation (22.4 mol%) (Table 3). The cyclic structure of the norbornene-based comonomers was found to attenuate *β*-H elimination and the related chain-walking process, while sulfur vulcanization of the unsaturated C=C bond-containing copolymers imparted greater tensile strength and improved toughness. Collectively, these studies show that [P,O]Pd catalysts can access a broad range of polar monomers beyond acrylates, with ligand rigidity and comonomer structure offering complementary handles to tune copolymer properties.

**Scheme 9:**
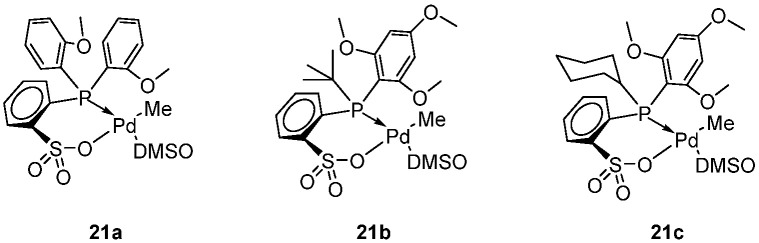
[P,O]-type Pd catalyst **21**.

In parallel, Zong et al. [67] described the copolymerization of ethylene with three bio-derived 2,7-octadienyl ether monomers using catalysts **21a–c** (Scheme 9). Studies have shown that only at low monomer concentration of 0.1 mol/L was the catalyst able to reach its maximum activity (10^4^ g/(mol·h)). In all cases, relatively low polar monomer incorporation (2.9 mol%) was obtained with complex **21b** and moderate copolymer MW (5.6 × 10^4^ g/mol) with complex **21c** (Table 3). The rates of chain propagation and chain transfer appeared to be greatly affected by the steric hindrance of the ligand: **21c** bearing bulky substituents with increased the axial steric hindrance to enhance the polymer MW by suppressing *β*-H elimination, but also suppressed the coordination of the polar unit to the Pd center.

**Scheme 10:**
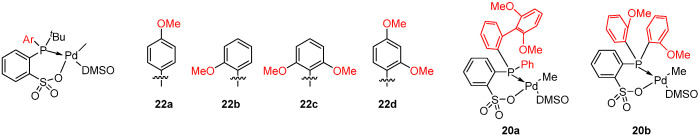
[P,O]-type Pd catalyst **22** and **20**.

In the design of [P,O]-type ligands, modifying substituents attached to the phosphorus atom remains the preferred strategy. In 2024, Liu et al. [68] investigated a series of phosphine-sulfonate palladium catalysts **22a**–**d** bearing tBu group on the phosphorus and electron-donating OMe on P-aryl substituents (Scheme 10), for E-NB or E-polar monomer copolymerization. The catalytic performance exhibited a strong dependence on both the position and number of OMe groups: increasing the number of *ortho*-OMe from one to two significantly increased catalytic activity, reaching a maximum of 1.0 × 10^6^ g/(mol·h) in the copolymerization of ethylene with polar-substituted norbornene derivatives. However, **22d** with a *para*-OMe on the P-aryl moiety exhibited enhanced activity but reduced the MW, suggesting that the steric hindrance from the *ortho*-OMe substituent not only restricts norbornene insertion but also suppresses chain-transfer reactions. Building on this insight that *ortho*-OMe steric bulk favorably modulates catalytic performance, Meng et al. [69] intentionally increased the steric bulk of substituents on the phosphorus atom in catalysts **20a–b** (Scheme 10), applied in the copolymerization of ethylene with fluorinated norbornene monomers. With a steric bulky architecture, catalyst **20a** maintains high activity at 10^6^ g/(mol·h) and superior comonomer incorporation ratios up to 28.9 mol%, outperforming catalyst **20b** (Table 3).

[P,O]-ligated Pd catalysts can catalyze the copolymerization of ethylene with a variety of commodity polar monomers, including readily available and bio-renewable ones, further expanding the range of polar feedstocks. In addition, this type of catalyst is characterized by generating essentially linear F-PE with polar monomer units situated in the backbone of the copolymers. However, more efforts are needed to develop appropriate systems that can generate high-MW copolymers (MW over 10^4^ g/mol).

**Table 3:** Ethylene-polar monomers copolymerization using [P,O] Pd catalysts.

Entry	Cat.	Comon.	t (°C)	Act. *^a^*	X_m_ *^b^* (%)	M_n_ *^c^*	PDI *^c^*	B *^d^*	T_m_ *^e^* (°C)	Ref.
1	**16b**	MA	85	0.29	2.3	0.11	1.8	-	83/105	[63]
2	**17**	MA	85	3.25	17	0.38	1.5	-	-	[63]
3	**18b**	6-Cl-Hex	80	101	5.3	0.29	2.8	2	103.5	[64]
4	**19**	^*t*Bu^NBE	120	0.54	3.5	2.4	2.9	-	88.4/109.1	[65]
5	**20a**	*trans*-NB-ole	80	138	2.7	18.8	1.68	-	99.9	[66]
6	**20b**	*trans*-NB-ole	80	204	19.4	7.4	1.73	-	-	[66]
7	**20b**	*trans*-NB-cin	80	165	22.4	6.8	1.49	-	-	[66]
8	**21a**	OC8-FUR	90	5.35	0.2	1.18	1.86	5.6	124.3	[67]
9	**21b**	OC8-SOL	90	0.95	2.9	2.82	2.16	-	117.2	[67]
10	**21c**	OC8-SOL	90	1.24	0.6	5.6	1.68	-	118.3	[67]
11	**22a**	NB_COOMe_	80	19	35.5	8.5	1.71	-	92.3	[68]
12	**22b**	NB_COOMe_	80	5.9	23.2	18.2	1.69	-	49.5	[68]
13	**22c**	NB_COOMe_	80	5.9	16	15.8	1.55	-	20.7	[68]
14	**22d**	NB_COOMe_	80	10.4	19.6	20.2	2.04	-	40.6	[68]
15	**20b**	Mono-NBF4	80	160	4.9	28	1.98	-	89.5	[69]

*^a^* Activity = 10^4^ g/(mol·h). *^b^* X_m_ = comonomer incorporation (mol %). *^c^* determined by GPC, ×10^4^ g/mol, PDI as polydispersity index. *^d^* B = branching density: branches/1000 C, the terminal functional groups are added to the total branches. *^e^* Determined by differential scanning calorimetry (DSC).

### 2.3. Other-Type Ligands

**Scheme 11:**
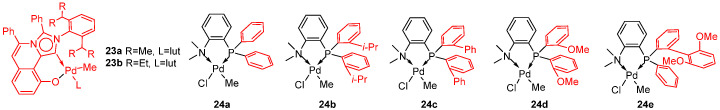
Other-type Pd catalysts **23**–**24**.

The direct relationship between ligand structure and olefin polymerization activity remains incompletely understood, prompting exploration of ligand types beyond *α*-diimine and [P,O] frameworks. In one example, Park et al. [70] developed Pd complexes **23a**–**b** bearing abnormal imidazo [1,5-a]pyridine-based N-heterocyclic carbene ligands (Scheme 11) and applied them in the copolymerization of ethylene with polar monomers (MA, MMA, BVE, VAc, allyl chloride (AC), *t*BA, and allyl ethyl ether (AEE); Table 4). The strong σ-donating ability of the ligand rendered the Pd center less electrophilic, thus reducing coordinated or inserted polar group back-donation and, in turn, increasing polar monomer incorporation. These catalysts also exhibited excellent thermal stability, and the produced copolymers had high MW. In the E-MMA copolymerization, the comonomer was incorporated into the main chain, without the formation of the enolate-terminated PE, which was attributed to 2,1-insertion of MMA. In a related study, Akita et al. [71] proposed a salt effect as the origin of increased methyl acrylate incorporation, where lithium borate salt accelerated chain transfer after ethylene insertion.

Further expanding the scope of non-classical ligands, Cao et al. [72] prepared a new family of phosphinobenzenamine Pd complexes **24a**–**e** bearing different groups at the *ortho*-position of P-aryl group (Scheme 11) for the copolymerization of E-MA or 5-hexene-1-yl-acetate (HAc). The maximum comonomer incorporation is 1.85 mol% with catalytic activity of 1.2 × 10^4^ g/(mol·h). While **24a**–**d** rely on either steric or electronic modulation separately, **24e** simultaneously tunes both effects, offering superior activity and a slower chain-walking process (Table 4).

**Table 4:** Ethylene-polar monomers copolymerization using other-type Pd catalysts.

Entry	Cat.	Comon.	t (°C)	Act. *^a^*	X_m_ *^b^* (%)	M_n_ *^c^*	PDI *^c^*	B *^d^*	T_m_ *^e^* (°C)	Ref.
1	**23a**	AC	100	0.18	3.0	0.54	2.0	-	109	[70]
2	**23b**	MA	100	0.4	2.3	1.4	1.3	-	112	[70]
3	**24b**	HAc	60	0.42	1.19	-	-	-	-	[72]
4	**24c**	HAc	60	0.60	0.48	0.65	3.27		-	[72]
5	**24e**	MA	60	1.2	-	0.77	2.6	-	100.8/113.6	[72]
6	**24e**	HAc	60	1.14	1.85	0.55	3.58	-	94.7/105.9	[72]

*^a^* Activity = 10^4^ g/(mol·h). *^b^* X_m_ = comonomer incorporation (mol %). *^c^* determined by GPC, ×10^4^ g/mol, PDI as polydispersity index. *^d^* B = branching density: branches/1000 C, the terminal functional groups are added to the total branches. *^e^* Determined by differential scanning calorimetry (DSC).

## 3. Ni Catalysts

Compared with Pd metal complexes, Ni is naturally abundant, economic, and possesses lower electronegativity, lower redox potentials, and stable low-valent Ni species. These properties made Ni catalysts widely used in oxidative addition or insertion reactions in organic chemistry [73], and Ni complexes have also been extensively studied for copolymerization of olefins with polar monomers. In addition, the lower electronegativity of Ni imparts greater persistence of alkyl-Ni intermediates toward *β*-H elimination [74,75,76]. Overall, the ligand type, steric and electronic tuning, secondary coordination sphere effects, and multinuclear strategies have all been shown to impact catalyst performance and are discussed in the following sections.

### 3.1. [N,N]-Type Ligands

**Scheme 12:**
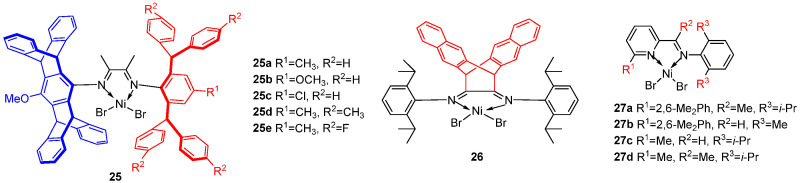
[N,N]-type Ni catalysts **25**–**27** with various modifications in ligand.

As an entry point into Ni-catalyzed copolymerization, unsymmetrical *α*-diimine ligands have been explored to tune electronic and steric effects. Hu et al. [77,78] reported a group of Ni complexes **25a**–**e** bearing unsymmetrical *α*-diimine structure (Scheme 12) for the copolymerization of E-MU using AlEt_2_Cl as activator (Table 5). Both catalytic activity and copolymer MW increased with the number of electron-donating methyl groups on the N-aryl rings. Among these structurally similar catalysts, **25d** approached maximum values for both activity and MW, confirming the positive role of weak electron-donating -Me groups. In contrast, **25c** bearing electron-withdrawing -Cl substituents showed moderate activity (similar to **25b**, 5.4 × 10^4^ g/(mol·h)), while **25e** failed to catalyze the copolymerization entirely. Complementing this electronic tuning strategy with a steric approach, Zhong et al. [79] designed dinaphthobarrelene-derived *α*-diimine Ni complex **26** (Scheme 12), which creates a three-dimensional constrained microenvironment around the Ni center. The bulky dinaphthobarrelene substituent fully shields the axial and back sites of the Ni center, preventing detrimental interactions with polar groups or counter anions and thereby enhancing both thermal stability and polar group tolerance (Table 5). Complex **26** showed good activity (up to 2.84 × 10^5^ g/(mol·h) at 20 °C) and remained stable at temperatures up to 80 °C for E-11-hydroxy-1-undecene (HU) copolymerization, producing copolymers with high MW (M_n_ up to 1.33 × 10^5^ g/mol), moderate comonomer incorporation (5.21 mol%), and notably narrow PDI (1-2). These two studies illustrate the complementary roles of electronic tuning and steric shielding in optimizing *α*-diimine Ni catalysts for polar monomer copolymerization.

Beyond *α*-diimine systems, pyridylamino Ni complexes represent another widely studied class for olefin polymerization [80,81,82,83]. Saki et al. [84] explored complexes **27a**–**d** bearing pyridylamino ligand (Scheme 12) for E-MA copolymerization. In the presence of AlEt_2_Cl, all complexes displayed moderate activities (up to 1.5 × 10^4^ g/(mol·h)) and produced low-MW, highly branched functionalized polyolefin oils with polar monomer content ranging from 0.2 to 35 mol%. DFT calculations on chain propagation, termination, and chain isomerization in ethylene polymerization [85] showed good agreement with experimental data, indicating that *ortho*-substitution on the pyridine moiety destabilizes ethylene coordination, thereby facilitating chain transfer and chain walking.

**Scheme 13:**
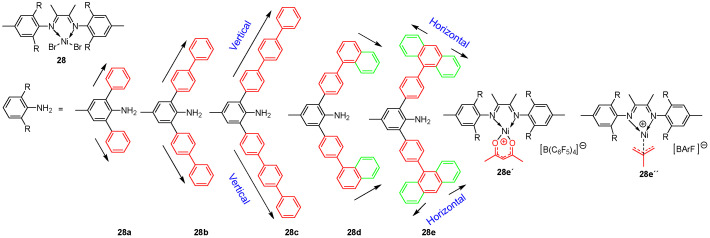
*α*-diimine Ni catalyst **28** adjusting horizontally and vertically steric in ligand.

Building on the recognition that steric tuning offers greater versatility than purely electronic modulation, Hu et al. [86] developed *α*-diimine Ni catalysts **28a**–**e** (Scheme 13) using a concerted strategy of simultaneously adjusting horizontal and vertical steric hindrance. In E-MU copolymerization, catalyst **28e″** showed very high activities (up to 2.64 × 10^6^ g/(mol·h)) and generated copolymer with high MW (M_n_ up to 1.32 × 10^5^ g/mol) and low comonomer incorporation (Table 5). These catalysts behaved similarly in copolymerization, except for complexes **28a**–**c**, which possess a vertically unidirectional steric environment and exhibited an order of magnitude lower activity with slightly reduced copolymer MW compared to **28d**–**e″**. This was attributed to the concerted horizontal and vertical steric bulk simultaneously inhibiting chain transfer and polar monomer insertion. Despite these advances, achieving higher polar monomer incorporation remains a central challenge in *α*-diimine Ni catalyst development.

**Scheme 14:**
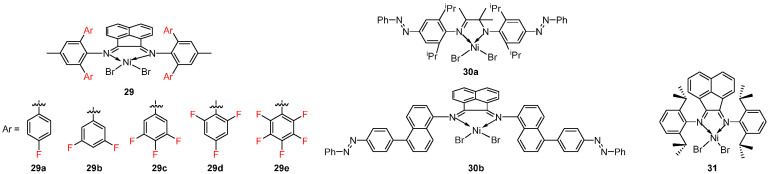
Ni catalysts 29–31 with various modifications in ligand.

Extending the scope of substituent effects, Hu et al. [87] have also investigated a group of complexes, **29a**–**e**, bearing *ortho*-, *meta*-, and *para*-fluorine-substituted *α*-diimine Ni complexes (Scheme 14) for copolymerization of ethylene with several polar monomers, including MU, UOH, UA, and VTEoS. When activated with AlEt_2_Cl, complexes **29a**–**e** showed high activities (up to 10^5^) and produced high-MW copolymers (M_n_ up to 10^5^ g/mol) (Table 5). The catalytic properties of **29** were similar to those of the previously discussed **28**, both producing copolymers with low polar monomer incorporation, suggesting that the fluorine substitution pattern offers no decisive advantage over the concerted steric strategy in enhancing comonomer uptake.

In parallel with ligand modification, external stimuli have emerged as a complementary strategy to regulate catalyst properties without altering the catalyst structure. Peng et al. [88] studied two *α*-diimine catalysts **30a**–**b** (Scheme 14) for E-MU copolymerization under ultraviolet light irradiation. This light-induced modulation of the ligand electronic effect resulted in slightly lower catalytic activity and produced copolymers with low comonomer incorporation (Table 5). Wang et al. [89] took a different approach, employing Bu_2_Mg as a stimulus/cocatalyst with complex **31** (Scheme 14) to catalyze the copolymerization of ethylene with several polar monomers such as MU, UA, and 10-undecenol. The reaction of Et_2_AlCl with Bu_2_Mg generated a solid MgCl_2_-supported Ni catalyst, yielding higher MW copolymers (up to 2.25 × 10^5^ g/mol) at 30 °C. In yet another approach, Tan et al. [90] synthesized novel metal-salt-based polar comonomers by reacting acidic polar monomers with alkyl metal reagents, which were then subjected to copolymerization with ethylene mediated by **31**. This system exhibited significantly higher catalytic activity (up to 8.2 × 10^5^ g/(mol·h)), good thermal stability (active at 90–150 °C), and produced a high-MW copolymer with relatively high comonomer incorporation (9.6 mol%) (Table 5). Compared to their steric analogues, the metal-salt-based comonomers exerted a weaker poisoning effect on the Ni center, contributing to the improved catalytic performance. Among these external-stimulus strategies, the metal-salt-based comonomer approach with **31** achieves the best combination of activity, thermal stability, and polar monomer incorporation, though it requires an additional comonomer pre-activation step.

[N,N]-type Ni catalysts have demonstrated the ability to achieve direct coordination copolymerization of ethylene with a variety of polar monomers, with axial steric hindrance in the ligand serving as a key factor for obtaining high-MW copolymers. However, the reliance on high-cost cocatalyst activation and the persistently low polar monomer content in the resulting copolymers remain critical issues that continue to drive research in this area.

**Table 5:** Ethylene-polar monomers copolymerization using [N,N] Ni catalysts.

Entry	Cat.	Comon.	t (°C)	Act. *^a^*	X_m_ *^b^* (%)	M_n_ *^c^*	PDI *^c^*	B *^d^*	T_m_ *^e^* (°C)	Ref.
1	**25a**	MU	30	19.2	0.06	16.9	1.56	147	-	[77]
2	**25b**	MU	30	5.4	0.1	11.3	1.41	138	-	[77]
3	**25c**	MU	30	3.8	0.06	7.35	1.66	151	-	[77]
4	**25d**	MU	30	38.9	0.11	27.3	1.37	150	-	[77]
5	**26**	HU	80	1.3	5.21	1.3	1.75	79	-	[79]
6	**28g**	MU	60	264	0.16	13.2	1.37	13.9	96.1	[86]
7	**29d**	MU	30	92	0.02	41.4	1.19	12.4	112.4	[87]
8	**30a**	MU	-	0.4	0.6	44.6	2.67	57	108.9	[88]
9	**30b**	MU	-	4.9	2.0	25.9	3.13	27	116.9	[88]
10	**31**	MU	30	4.2	3.2	22.5	4.5	-	122.5	[89]
11	**31**	MU-Al	40	82	9.6	16	2.5	80	-	[90]
12	**31**	MU-Al	90	42	36	3.03	3.8	99	-	[90]

*^a^* Activity = 10^4^ g/(mol·h). *^b^* X_m_ = comonomer incorporation (mol %). *^c^* determined by GPC, ×10^4^ g/mol, PDI as polydispersity index. *^d^* B = branching density: branches/1000 C, the terminal functional groups are added to the total branches. *^e^* Determined by differential scanning calorimetry (DSC).

### 3.2. [N,O]-Type Ligands

[N,O]-ligated Ni(II) complexes have expanded the scope of ligand design beyond [N,N] systems, providing additional opportunities for efficient copolymerization of polar monomers. The salicylaldimine ligands reported by Grubbs et al. [24,91,92] demonstrated the utility of [N,O]-type Ni catalysts for ethylene copolymerization with polar norbornene derivatives, while β-ketoiminato catalysts developed by Li, Chen, and co-workers [93,94] successfully catalyzed the copolymerization of ethylene with MMA. Despite these early successes, these catalysts still suffered from low thermal stability, low copolymer MW, and limited tolerance toward a broader scope of polar monomers. Nevertheless, [N,O]-ligated Ni catalysts provide an attractive platform to combine diverse ligand structural tuning strategies for achieving superior catalyst performance, and subsequent studies have focused on ligand backbone modification and functional group modulation.

**Scheme 15:**
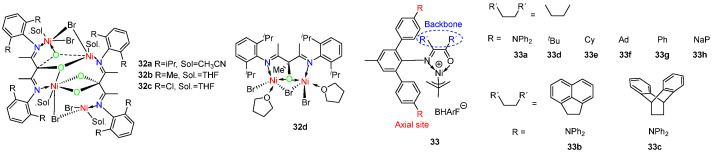
Multinuclear Ni catalyst **32** and catalyst **33** adjusting by sterically hindrance.

Ethylene copolymerization with industrially relevant short-chain alkenoic acids has attracted increasing attention in recent years. In this context, Ji and co-workers [95] reported a new family of tetranuclear Ni complexes **32a**–**c** and a binuclear complex **32d** (Scheme 15) for direct copolymerization of ethylene with various polar comonomers, including AA, vinyl acetic acid (VA), and methyl vinyl acetate (MVA). Complex **32a** exhibited clear signs of living copolymerization and afforded moderate-MW copolymers (2.83 × 10^4^ g/(mol·h)) with high branching densities (76/1000 C). Compared with the tetranuclear **32a–c**, the binuclear complex **32d** with a shorter Ni⋯Ni distance showed superior performance for the copolymerization of ethylene with VA and AA, which was ascribed to the distinctive vinyl acetic acid enchainment enabled by Ni⋯Ni synergistic effect, providing an additional mode of polar monomer incorporation (Table 6). This work highlights the potential of multinuclear cooperativity as a design principle distinct from the mononuclear ligand-tuning strategies discussed previously.

The effect of backbone and axial substituent in α-imino-ketone Ni complexes **33a–h** was investigated by Chu et al. (Scheme 15) [96]. The results showed that the butane-based ligand backbone was beneficial for improving the overall catalytic performance (activity, MW, branching density, and comonomer incorporation), while there was no explicit evidence for a significant influence of axial substituents on the copolymerization.

**Scheme 16:**
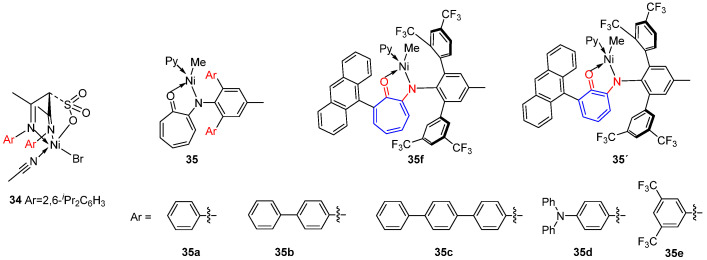
Ni catalysts **34**–**35** bearing different steric hindrance substituents.

A significant advance for [N,O] Ni catalysts was reported by Du et al. [97], who designed a new Ni catalyst **34** bearing an *α*-sulfonato-*β*-diimine ligand for copolymerization of E-MA (Scheme 16). This single-component catalyst **34** displayed good catalytic activity, and increasing the MA concentration enhanced the incorporation of MA in the copolymer. Notably, **34** generated copolymers with both MA incorporation greater than 10 mol% and M_n_ higher than 2 × 10^4^ g/mol, representing a successful breakthrough for [N,O]-ligated Ni catalyst systems in simultaneously achieving high polar monomer content and high MW. The introduction of the sulfonate group reduced the oxophilicity of the Ni center. To further elucidate the mechanism leading to terminal MA units in the copolymer, DFT calculations showed that 2,1-insertion of MA is thermodynamically favorable among the transition state intermediates, followed by subsequent *β*-H elimination to generate terminal MA units.

Further expanding the [N,O] ligand family, Li et al. [98] synthesized neutral anilinotropone Ni catalysts **35a–f** bearing different steric substituents at the *ortho*-positions of the N-aryl moiety (Scheme 16). In the absence of an activator, these Ni catalysts exhibited moderate activities and good thermal stability (stable at 70 °C) in copolymerization of ethylene with 5-hexene acetate (HAc). The copolymers were characterized by high MW (~10^5^ g/mol) with a narrow PDI 1-2. The effect of the substituents on the catalyst properties showed no clear trend; notably, catalyst **35f**, bearing a similar structure to the conventional salicylaldehyde Ni catalyst **35′**, achieved higher MW and higher comonomer incorporation (Table 6).

**Table 6:** Ethylene-polar monomers copolymerization using [N,O] Ni catalysts.

Entry	Cat.	Comon.	t (°C)	Act. *^a^*	X_m_ *^b^* (%)	M_n_ *^c^*	PDI *^c^*	B *^d^*	T_m_ *^e^* (°C)	Ref.
1	**32a**	VA	30	8.4	0.4	2.83	3.6	76	99	[95]
2	**32d**	VA	30	46.8	0.5	5.88	3.5	78	24	[95]
3	**33a**	MU	30	17.53	2.9	1.34	2.5	12.6	-	[96]
4	**34**	MA	50	0.26	10.2	2.03	1.12	45	91.1	[97]
5	**35e**	HAc	70	9	0.11	16.2	1.34	44	99	[98]
6	**35f**	HAc	65	15.7	0.25	23.7	1.92	19	110	[98]
7	**35′**	HAc	65	3.5	0.16	0.7	2.03	26	106	[98]
8	**36a**	MU	25	95.4	0.71	73.2	2.33	25	127.58	[99]
9	**36b**	MU	25	111.3	0.56	11.6	2.87	17	119.96	[99]
10	**37a**	MU	40	9.83	0.21	0.19	1.4	-	113.4	[100]
11	**38b**	UA	40	24.9	0.13	43.9	2.67	-	-	[101]

*^a^* Activity = 10^4^ g/(mol·h). *^b^* X_m_ = comonomer incorporation (mol %). *^c^* determined by GPC, ×10^4^ g/mol, PDI as polydispersity index. *^d^* B = branching density: branches/1000 C, the terminal functional groups are added to the total branches. *^e^* Determined by differential scanning calorimetry (DSC).

**Scheme 17:**
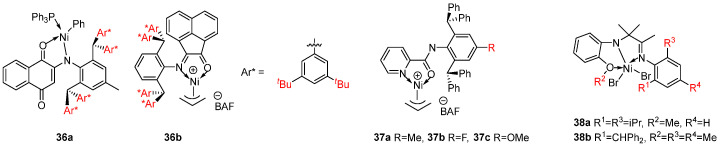
[N,O]-type Ni catalysts **36**–**38**.

Continuing the exploration of sterically modified [N,O] Ni catalysts, Chen and co-workers [99] reported complexes **36a**–**b** bearing eight *tert*-butyl groups in diphenyl-methyl on *ortho*-positions of N-aryl moiety (Scheme 17). These complexes exhibited high solubility in aliphatic hydrocarbons and displayed high catalytic activity up to 10^6^ g/(mol·h) in E-MU copolymerization. Meanwhile, Li et al. [100] showed that the catalytic activity of complexes **37a**–**c** bearing *para*-substituents on the N-aryl group (Scheme 17) followed the order **37a** (R = F) > **37b** (R = Me) > **37c** (R = OMe), attributed to electronic effects imparted by the R substituents. Adding a new structural dimension, Jiang et al. [101] investigated complexes **38a**–**b** bearing different steric substituents on the N-aryl group (Scheme 17) for copolymerization of ethylene with different polar monomers (MU, VA, 6-Cl-Hex, and 10-undecylenic alcohol). A distinguishing feature of **38a**–**b** is that secondary interactions between the O and Ni atoms can influence catalytic performance (Scheme 17). Upon activation with AlEt_2_Cl, catalysts **38** exhibited moderate catalytic activities (up to 10^5^ g/(mol·h)) and afforded high-MW copolymers (up to 10^5^ g/mol). The introduction of *^i^*Pr and Me at the *ortho*-position of the N-aryl groups broadened the scope of compatible polar monomers. Together with **36** and **37**, these studies demonstrate that steric bulk, electronic tuning, and secondary interactions each contribute to optimizing the performance of [N,O] Ni catalysts for polar monomer copolymerization.

Taking together, these results indicate that most [N,O]-ligated Ni catalysts can operate without hazardous and costly alkyl-aluminum cocatalysts or scavengers while still producing high-MW copolymers. This feature, combined with the structural diversity accessible through backbone and substituent modification, reinforces [N,O] systems as a versatile platform for polar monomer copolymerization.

### 3.3. [P,O]-Type Ligands

[P,O]-ligated Ni(II) catalysts have long been recognized for their exceptional tolerance to polar functional groups and high catalytic activity. One of the most successful [P,O] Ni-based systems, the SHOP (Shell Higher Olefin Process) catalyst, was first reported in 1987 [102] and is employed for the commercial oligomerization of ethylene to produce *α*-olefins. However, this catalyst is limited to copolymerization of ethylene with polar monomers bearing remote polar functionalities. A significant advance came with Xin et al. [103], who reported a modified Ni-SHOP-type single-component catalyst for direct copolymerization of ethylene with alkyl acrylates, yielding linear, high-MW copolymers with low to moderate acrylate incorporation. This work bridged the gap between classic SHOP-type systems and direct acrylate copolymerization, opening a new direction for [P,O] Ni catalyst development.

**Scheme 18:**
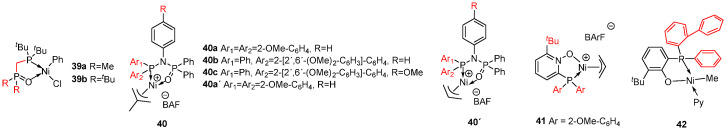
[P,O]-type Ni catalysts **42** with various modifications in ligand.

Further advances in [P,O] Ni catalysts focused on improving thermal stability and comonomer incorporation through both cocatalyst and ligand design. Jung et al. [104] reported that the methylene-bridged bisphosphine monoxide-ligated Ni catalyst **39** exhibited excellent thermal stability for E-AAc copolymerization (Scheme 18). When activated with MMAO at 80 °C, the activity could reach 10^3^ g/(mol·h) (Table 7). It was proposed that a portion of the Lewis acidic Al center in MMAO competes for binding with the carbonyl group of AAc, thereby reducing the catalyst poisoning effect caused by carbonyl back-biting. In a complementary approach targeting improved comonomer incorporation, Xu et al. [105] explored diphosphazane monoxide Ni complexes **40a**–**c** (Scheme 18) bearing different substituents for E-MA copolymerization in the absence of an activator. The 2-methylallyl Ni catalyst improved the chain initiation rate and effectively promoted the copolymerization reaction, thereby increasing comonomer incorporation to 7.0 mol% and catalytic activity to 10^4^ g/(mol·h). Moderate-MW copolymers (10^4^ g/mol) with high melting temperatures (up to 123.9 °C) were obtained, and ^1^H NMR spectroscopy revealed that MA was inserted both at the chain end and in the main chain. Together, these studies demonstrate that [P,O] Ni catalysts continue to advance toward practical applications by addressing key limitations: Jung’s system tackles thermal stability, while Xu’s work improves polar monomer incorporation without sacrificing catalyst activity.

Ligand design in [P,O] Ni systems has also been directed toward controlling branching and expanding the scope of compatible polar monomers. Zhang et al. [106] studied a 2-phosphino-pyridine-N-oxide Ni complex **41** (Scheme 18) for E-MU copolymerization. **41** produced lightly branched copolymers with low MW (1.43 × 10^3^ g/mol), moderate comonomer incorporation, and high activity up to 3.5 × 10^5^ g/(mol·h) at 90 °C (Table 7). Temperature had a pronounced effect: decreasing to 30 °C reduced the catalytic activity by an order of magnitude and lowered comonomer incorporation to 0.4 mol%. In a separate study targeting different polar monomers, Zhang and co-workers [107] reported a phosphinophenolate Ni complex **42** bearing a bulky alkyl group on the phosphorus atom (Scheme 18), which helps to suppress *β*-H elimination. Under activator-free conditions, this bulky Ni catalyst promoted the copolymerization of ethylene with vinyl sulfones (VS) with satisfactory results (Table 7). Owing to the preference of vinyl sulfone for 2,1-insertion into Ni-CH_3_ and its strong coordination ability, *β*-H elimination was inhibited, resulting in lower catalytic activity and copolymer MW compared to those from E-MA copolymerization. These studies highlight that while steric bulk on phosphorus can effectively suppress chain transfer, the choice of comonomer introduces additional coordination effects that must be carefully balanced against catalyst activity.

**Scheme 19:**
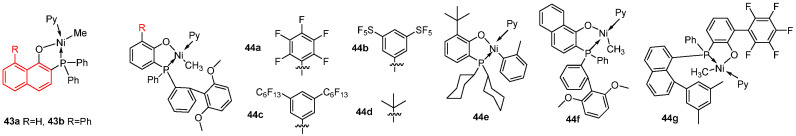
[P,O]-type Ni catalysts **43**–**44** with various modifications in ligand.

Further exploring steric tuning on the phosphorus atom, Wang et al. [108] developed an approach to optimize the catalyst structure through the phosphino-naphtholate framework bearing steric bulky substituents at the C-8 position. The bulky substituents in catalysts **43a**–**b** (Scheme 19) crowded the space around the Ni center while reducing the phosphine chelating ability. Notably, **43a**–**b** successfully copolymerized ethylene with the extremely challenging 1,1-disubstituted difunctional comonomer dimethyl itaconate (DMI), achieving catalytic activity of 3.08 × 10^4^ g/(mol·h) and producing copolymers with end-difunctional diester groups, albeit with low MW (4.86 × 10^3^ g/mol). In E-MA copolymerization, **43b** showed higher activity up to 4.05 × 10^5^ g/(mol·h) with slightly higher MA incorporation but similarly low MW (Table 7). This study demonstrates that carefully tuned steric bulk on phosphorus can unlock access to challenging 1,1-disubstituted comonomers, expanding the monomer scope of [P,O] Ni catalysts beyond conventional vinyl monomers.

Building on the expanded monomer scope, Baur et al. [109] reported that [P,O]-type Ni catalysts **44a**–**g** (Scheme 19) bearing bulky substituents on the phenolate moiety enabled non-alternating copolymerization of ethylene with carbon monoxide. This strategy successfully incorporates low-density, isolated in-chain ketone groups into high-MW PE chains (M_n_ up to 2.16 × 10^5^ g/mol). The resulting copolymers exhibit tensile properties comparable to commercial HDPE while simultaneously demonstrating photo-degradability, positioning them as a promising sustainable alternative for addressing plastic waste challenges.

**Scheme 20:**
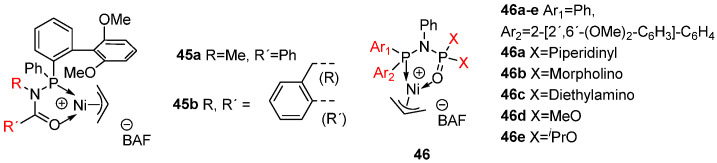
[P,O]-type Ni catalysts **45**–**46** with various modifications in ligand.

Research on modifying substituents on the ligand backbone has also proven effective for improving [P,O]-type Ni catalyst performance. Zhu et al. [110] recently studied the impact of steric and electronic effects of backbone substituents on phosphine carbonyl Pd and Ni catalysts. **45a**–**b** (Scheme 20) bearing a ring structure and methyl groups on the ligand backbone expanded the substrate scope of polar monomers (MA, AAc, and AC), with catalytic performance comparing favorably to previous [P,O]-type Ni catalysts (Table 7). In a related strategy applied to phosphoramide ligands, **46a**–**e** (Scheme 20) bearing substituents with different steric and electronic properties [111] were shown to mediate ethylene copolymerization with a variety of polar monomers, including MA, 6-Cl-Hex, MU, methyl 5-norbornene-2-carboxylate, and vinyltrimethoxysilane, in the absence of any cocatalyst, protecting reagents, or scavengers. Notably, catalyst **46d** bearing fewer electron-donating P(O)(OR)_2_ groups significantly improved catalytic activity without sacrificing other performance metrics. These backbone modification studies demonstrate that electronic and steric tuning at positions remote from the metal center can still exert substantial influence on catalyst performance, offering an additional design parameter for [P,O] Ni catalyst optimization beyond substituent changes on phosphorus or oxygen.

**Scheme 21:**
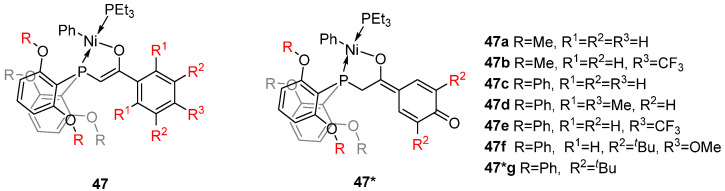
Ni catalyst **47** bearing large phosphine substituents.

Turning to ligand architecture with axial steric protection, Xiong et al. [112] found that neutral Ni phosphine enolate catalysts **47a–g** (Scheme 21) having bulky phosphine substituents located at the axial positions showed high efficiency for the copolymerization of ethylene with acrylate in terms of catalytic activity and thermal stability. Among these, **47c**, **e–f** showed activities up to 10^6^ g/(mol·h) at 70–90 °C with extra Ni(COD)_2_ as the triethylphosphine scavenger and produced moderate-MW copolymers with low *t*BA incorporation, while **47c** had the best thermal stability, with activity reaching 7.7 × 10^6^ g/(mol·h) even at 110 °C (Table 7). The axial shielding provided by the *ortho* phenoxy group on the phosphine was found to be a prerequisite for high activity and high-temperature performance of the catalytic system. Although the copolymer MW and monomer incorporation are not outstanding in this work, this axial shielding strategy is undoubtedly promising for achieving high-temperature E-polar monomer copolymerization.

**Scheme 22:**
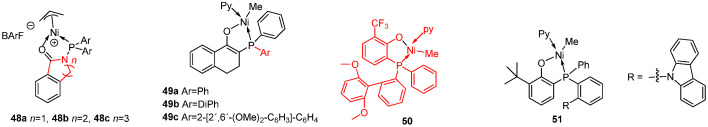
[P,O]-type Ni catalysts **48**–**51** with various modifications in ligand.

**Scheme 23:**
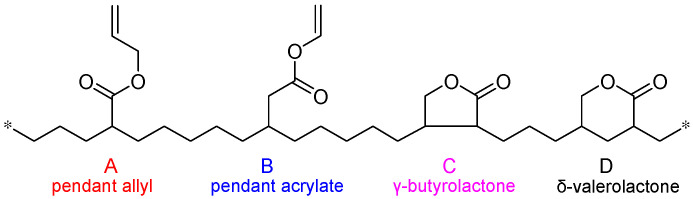
Complex **50** catalyzed copolymerization of ethylene with allyl acrylate to generate a multiplex copolymer structure.

Ligand backbone ring-size has also been explored as a strategy to tune [P,O] Ni catalyst performance. Cui et al. [113] proposed N-bridged phosphine-carbonyl Ni catalysts **48a**–**c** (Scheme 22) in which the ring-size of the N-containing bridge governed catalytic behavior. The results demonstrated that the seven-membered-ring bridge (n = 3) gave the highest E-MA copolymerization performance, with activity decreasing as the ring size was reduced from eight- to five-membered-ring structures (Table 7). Applying a similar strategy with steric modification on phosphorus, catalysts **49a**–**c** (Scheme 22) [114] tended to increase the incorporation of polar monomers into the copolymer backbone, with MA incorporation reaching up to 8.2 mol%. In a related study targeting more complex copolymer architectures, Wang et al. [115] investigated a phosphinophenolate complex **50** (Scheme 22) bearing bulky substituents for E-AA (allyl acrylate) copolymerization. The resulting copolymers contained multiple structural units in the backbone (Scheme 23), including noncyclic units bearing pendant allyl moieties (A, insertion with the acrylic double bond), and pendant acrylate moiety (B, insertion with the allyl double bond), as well as cyclic units: *γ*-butyrolactones (C, incorporated with 1,2-acrylate insertion, followed by the allyl unit 2,1-insertion) and *δ*-valerolactone (D, incorporated with 1,2-acrylate insertion, followed by the allyl unit 1,2-insertion). These structurally diverse copolymers offer potential applications as blending compatibilizers or crosslinking agents for polyolefin materials, and the pendant double bonds can serve as sites for further functionalization.

In a further development, Wang et al. [116] employed catalyst **51** (Scheme 22) to coordinate with a Lewis acidic metal salt (Ni(OAc)_2_, Zn(OAc)_2_, or Zn(TMEDA)(OAc)_2_) to increase the steric hindrance around the metal center, significantly enhancing the MW of copolymers in ethylene-*t*BA copolymerization.

**Scheme 24:**
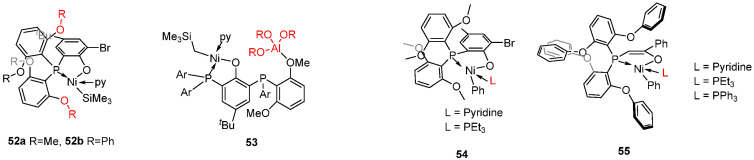
[P,O]-type Ni catalysts **52**–**55**.

Efforts to enhance catalytic activity and thermal stability were pursued by Xiong et al. [117] through complementary approaches. Bulky neutral Ni catalysts **52a**–**b** (Scheme 24) were developed for E-*t*BA copolymerization, demonstrating exceptional activity (up to 3.7 × 10^7^ g/(mol·h)) and thermal stability up to 130 °C under activator-free conditions (Table 7). This outstanding performance was attributed to the larger ligand framework lowering the residence time of acrylate-inserted transition-state species while also permitting acrylate-induced *β*-H elimination; however, further optimization is still needed to improve comonomer incorporation. The same group also investigated the effect of adding a neutral, rather than cationic, Lewis acidic metal to Ni catalyst **53** (Scheme 24) [118]. The Ni-Al heterobimetallic species generated in situ by mixing catalyst **53** with Al(OiPr)_3_ promoted the copolymerization of ethylene with *t*BA with high activity (up to 10^6^ g/(mol/h)) and polar comonomer incorporation up to 2.2 mol% (Table 7). Furthermore, choosing an appropriate labile ligand L (e.g., L = pyridine) in catalysts **54**–**55** [119,120] led to faster chain propagation and more efficient catalyst activation (activity up to 2.4 × 10^7^ g/(mol·h)) without significantly altering the copolymer microstructure (Scheme 24). Compared to the neutral bulky catalysts **52a**–**b**, the heterobimetallic and labile ligand strategies offer alternative routes to achieve high activity, though each involves a trade-off among comonomer incorporation, MW, and operational complexity.

**Scheme 25:**
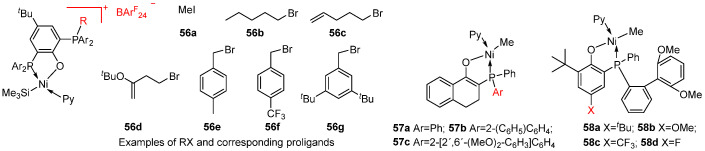
[P,O]-type Ni catalysts **56**–**58**.

Structural modifications of the ligand framework were explored by Ghana et al. [121] who synthesized catalysts **56a–g** (Scheme 25) for E-acrylate copolymerization through alkylation of pendant phosphine, followed by anion exchange. This methodology primarily investigated electrophilic reagents and anions in diverse late-stage functionalization approaches. Compared to Brookhart-type [N,N]Pd catalysts, these cationic catalysts enabled the production of copolymers with minimal methyl branching and vinyl chain ends. The advantageous role of electron-donating groups was further demonstrated in several subsequent studies. Catalyst **57c** (Scheme 25), bearing bulky substituents and electron-donating methoxy (−OMe) groups, enabled copolymerization with polar styrene derivatives with 1.32–4.16 mol% incorporation [122]. Similarly, phosphinophenolate Ni catalysts **58a–d** (Scheme 25) containing -OMe substituents successfully mediated ethylene copolymerization with UA, *t*BA, or 6-Cl-Hex (Table 7) [123]. Mechanistic analysis revealed that electron-withdrawing groups render the metal center more electrophilic, facilitating stable chelate formation with polar monomers but concurrently sacrificing catalytic activity. Overall, these studies underscore the critical balance between electronic modulation and catalytic performance in polar monomer copolymerization systems.

**Scheme 26:**
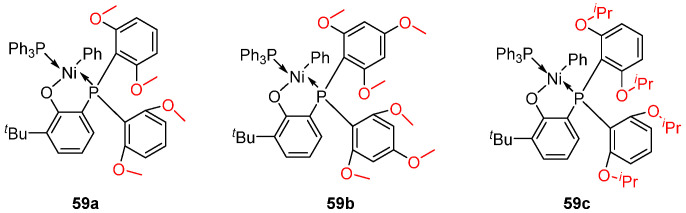
[P,O]-type Ni catalyst **59**.

Building on the advantageous role of OMe-substituted phosphinophenolate Ni catalysts, Liu et al. [124] systematically investigated electron-donating group-modified phosphinophenolate nickel catalysts (**59a–c**; Scheme 26) for aqueous coordination–insertion copolymerization of ethylene with various polar monomers (6-Cl-Hex, 5-hexen-1-ol, 4-penten-1-ol, 10-undecen-1-ol, hex-5-en-2-one, and hept-6-enyl acetate) under mild conditions. The nickel catalysts effectively overcame unfavorable interactions between polar monomers/water and active metal centers, enabling the production of linear polar PE with high MW (2.19–5.49 × 10^5^ g/mol) and controlled polar group incorporations (0.13–1.29 mol%). Notably, catalyst **59c** demonstrated superior performance in generating copolymers with elevated MW, prompting further detailed investigations [125]. When applied to ethylene-MA, *n*BA, or *t*BA copolymerization at 50 °C under 2 MPa ethylene pressure, **59c** directly yielded ultra-high-MW copolymers (M_n_ ranging from 1 × 10^6^ to 2.7 × 10^6^ g/mol), demonstrating the capability of this system to access exceptionally high-MW polar polyethylenes. Mechanistic studies elucidated the 2,1-insertion mode of acrylate monomers and identified critical dormant species during copolymerization, achieving an optimal balance among polymer MW, catalytic activity, and comonomer insertion efficiency (Table 7). This work establishes **59c** as one of the most effective [P,O] Ni catalysts for accessing ultra-high-MW polar polyethylenes, a long-standing challenge in the field.

For [P,O] Ni catalysts, the introduction of bulky substituents on the phosphorus atoms of the ligand is crucial for enhanced catalytic activity and the formation of high-MW linear copolymers. Large sterically hindered [P,O] neutral Ni complexes can achieve direct copolymerization of ethylene with commercially available polar vinyl comonomers, making them among the most promising catalysts compared to other Ni analogues.

**Table 7:** Ethylene-polar monomers copolymerization using [P,O] Ni catalysts.

Entry	Cat.	Comon.	t (°C)	Act. *^a^*	X_m_ *^b^* (%)	M_n_ *^c^*	PDI *^c^*	B *^d^*	T_m_ *^e^* (°C)	Ref.
1	**39**	AAc	80	0.24	0.51	0.23	2.9	-	-	[104]
2	**41**	MU	90	35	1.2	0.14	-	9	101	[106]
3	**41**	MU	30	3.4	0.4	0.29	-	9	116	[106]
4	**42**	PVS	70	0.25	6.1	1.21	1.2	-	113	[107]
5	**42**	MA	50	18.3	1.6	17.9	2.8	-	124	[107]
6	**43a**	DMI	70	3.08	0.79	0.48	2.2	-	126	[108]
7	**43b**	MA	70	40.5	2.7	0.06	1.8	-	112	[108]
8	**44a**	CO	100	7.82	1	21.6	1.8	-	136	[109]
9	**45b**	MA	80	4	1.1	1.37	1.8	-	125	[110]
10	**45b**	AAc	80	0.2	0.7	0.87	2.6	-	127	[110]
11	**45b**	AC	80	0.65	0.6	1.29	2.4	-	133	[110]
12	**46c**	MA	80	0.26	2.8	0.48	2.2	-	110.1	[111]
13	**46d**	MA	80	1.01	2.2	0.66	2.2	-	117.8	[111]
14	**47c**	*t*BA	110	770	0.4	0.38	2.5	-	123	[112]
15	**47e**	*t*BA	90	134	0.5	0.46	2.2	-	122	[112]
16	**47f**	*t*BA	70	185	0.6	0.66	2.3	-	123	[112]
17	**48b**	MA	30	0.04	1.4	0.14	-	3	95.5	[113]
18	**48c**	MA	30	0.33	3.1	0.098	1.83	7	104.5	[113]
19	**49**	MA	50	0.3	8.2	0.54	2.2	-	87	[114]
20	**51**	*t*BA	80	16	1.2	3.28	2.5	-	123.6	[116]
21	**52a**	*t*BA	130	3700	0.3	0.6	2.6	-	127	[117]
22	**53**	*t*BA	90	100	2.2	2.23	2.3	-	110	[118]
23	**57c**	p-VinylSt	50	2.1	4.16	0.65	1.9	-	114	[122]
24	**58b**	MA	80	2.4	0.7	1.8	2.2	-	123.5	[123]
25	**59c**	MA	50	220	0.32	270	1.62	-	127.6	[125]

*^a^* Activity = 10^4^ g/(mol·h). *^b^* X_m_ = comonomer incorporation (mol %). *^c^* determined by GPC, ×10^4^ g/mol, PDI as polydispersity index. *^d^* B = branching density: branches/1000 C, the terminal functional groups are added to the total branches. *^e^* Determined by differential scanning calorimetry (DSC).

## 4. Copolymerization Mechanism

Most Pd and Ni catalysts designed for copolymerization of ethylene with polar monomers have made considerable progress toward practical industrial application. The development of desirable catalysts is established based on a full understanding of the balance between structural evolution and practical performance. Furthermore, elucidating the copolymerization reaction mechanism is another key factor for the development of high-performance catalysts that simultaneously possess high catalytic efficiency, high polar monomer incorporation capability, and produce high-MW copolymers with controllable microstructure.

**Scheme 27:**
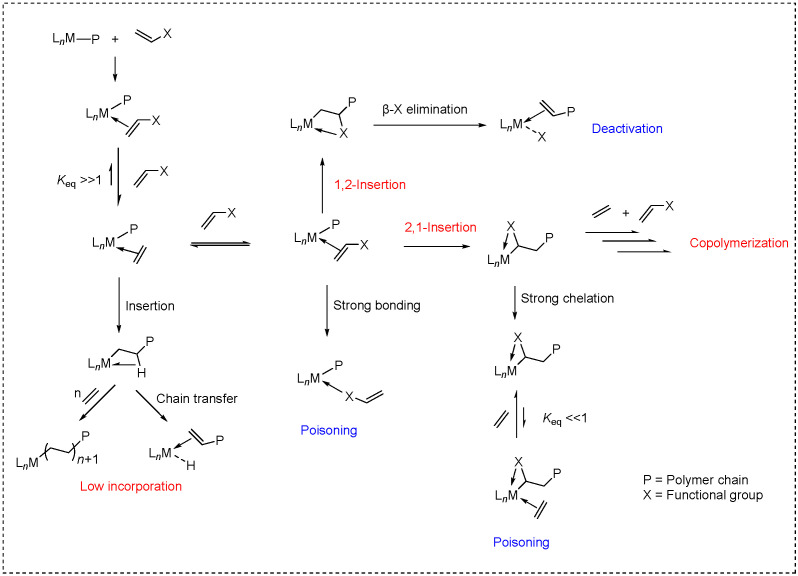
General mechanism of the copolymerization of ethylene with polar monomers.

For the mechanism of transition-metal-catalyzed ethylene copolymerization, key aspects that have been investigated through experimental and computational studies include catalyst deactivation pathways, polar monomer insertion modes, and chain-transfer reactions. The well-documented cause of catalyst poisoning is the “monomer problem”, in which the Lewis basic polar group containing oxygen and/or nitrogen atoms preferentially undergoes nucleophilic reaction with the Lewis acidic metal center. The 1,2-insertion or 2,1-insertion and subsequent isomerization of the polar monomer determine the position of the polar monomer unit in the PE chain. The relative rate of coordination insertion between vinyl polar monomers and ethylene is a key factor governing the monomer incorporation ratio, while the rate of chain termination processes, such as *β*-X elimination, determines the copolymer MW. Scheme 27 presents the copolymerization mechanism that has been established through extensive experimental and computational mechanistic studies (applicable to both Pd and Ni catalysts) [16,126,127,128]. Wang et al. [129] summarized the mechanism of ethylene copolymerization with polar monomers in 2023; in the following sections, we focus on new mechanistic insights supported by experimental or computational evidence reported in recent years.

**Scheme 28:**
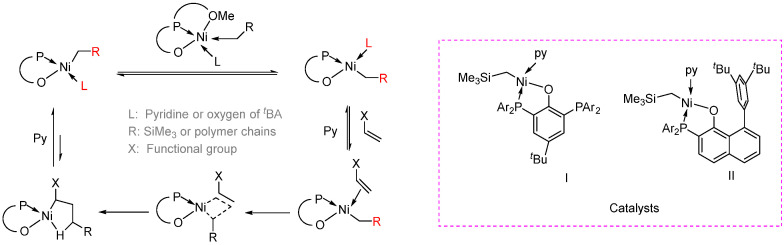
Mechanism of the ethylene-*t*BA copolymerization using neutral [P,O]-type Ni catalysts.

**Scheme 29:**
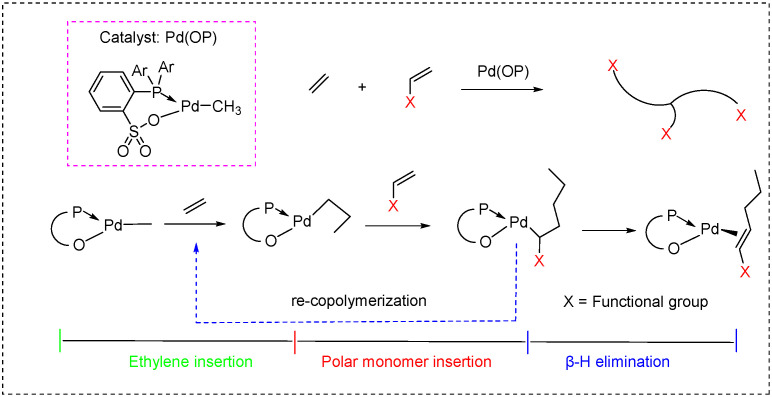
Mechanism of the insertion of the polar monomer into the PE chain.

Xiong et al. [130] investigated the mechanism of E-*t*BA copolymerization using neutral [P,O] Ni catalysts by a combination of kinetic studies and computational (DFT) methods. The results showed that the enchainment of acrylate and ethylene had similar rate constants, resulting in a propagation sequence in which *t*BA inserts first, followed by ethylene (Scheme 28). This was attributed to the unfavorable steric interaction between the *t*BA-inserted growing polymer chain and the bulky OMe side group of the ligand, which made the rate of ethylene incorporation after *t*BA insertion an order of magnitude slower than that after ethylene insertion. The site-isomerization process of the catalyst was also found to be essential, as switching the growing polymer chain from trans to O to trans to P provided a lower insertion barrier in the reaction pathway, facilitating the insertion of coordinated olefins in subsequent steps and enabling chain migration via 2,1-insertion mode. A similar catalytic mechanism has been identified for neutral phosphinosulfonate Pd catalysts in the copolymerization of E-2-methoxystyrene [131], with DFT calculations revealing that ethylene molecules preferentially coordinate to the Pd center trans to the P→Pd bond, which facilitates the insertion of the polar monomer through the 2,1-insertion pathway in chain propagation, followed by *β*-H elimination leading to in-main-chain incorporation of the polar monomer (Scheme 29).

**Scheme 30:**
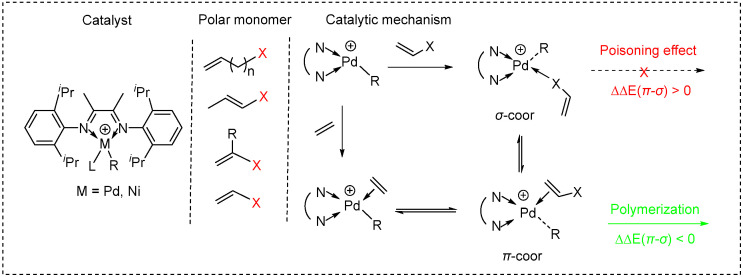
Mechanism of monomer poisoning effect in the copolymerization of ethylene and polar monomer.

In addition to insertion and chain-transfer pathways, the poisoning effect of polar monomers on the active metal center has been systematically investigated through computational methods. Zhao et al. [132] used the Brookhart-type catalysts to study the mechanism of monomer poisoning effect in the copolymerization of ethylene and polar monomer (Scheme 30). Based on DFT calculations and multivariate linear regression (MLR) analysis, the energy difference between double-bond coordination and heteroatom coordination was identified as the primary factor governing the poisoning effect. For polar monomers such as acrylonitrile, methylene-spaced vinyl esters, and internal olefins, the energy difference ∆∆E(π-σ) was greater than 0, indicating that the heteroatom complex is more stable than the double-bond complex and exhibits obvious poisoning behavior. In contrast, the poisoning effect was relatively weak for polar monomers such as alkenyl ethers, which exhibit smaller energy differences ∆∆E(π-σ) < 0.

**Scheme 31:**
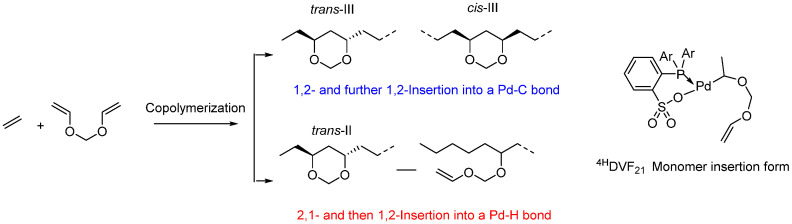
The steric effect mechanism of the Pd catalyst.

Computational studies have also elucidated how catalyst steric hindrance governs regioselectivity in copolymerization with difunctional monomers. Mehmood et al. [133] used the Gaussian 16 program to calculate the steric effect of Pd catalysts on olefin–divinyl formal (DVF) copolymerization (Scheme 31). The catalyst ^4H^DVF_12_, with the largest steric hindrance (buried volume of 85.7), favored the 1,2-insertion route for DVF insertion in the Pd-C bond, after which a stable six-membered acetal unit is prone to *β*-H elimination that terminates the copolymerization, leading to reduced catalytic activity and yielding a low-MW copolymer. In contrast, for the minimally hindered naphthalene-bridged (P,O)PdMe catalyst (buried volume of 47.8), the energy barrier of 2,1-DVF insertion (5.1 kcal mol^−1^) is lower than the 1,2-DVF insertion, ultimately facilitating subsequent monomer insertion.

**Scheme 32:**
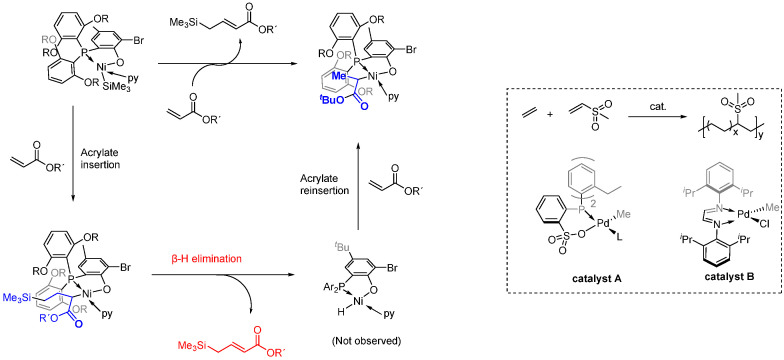
Mechanism of *β*-H elimination and the Pd catalyst steric effect. (Note: The left figure is from the study by Xiong et al., and the right figure is from the study by Zhu et al.)

Experimental kinetic studies have also provided insight into the role of *β*-H elimination in controlling copolymer microstructure. Xiong et al. [117] investigated the mechanisms of phosphinophenolate Ni catalysts and their *β*-H elimination behavior in copolymerization of ethylene with acrylate (Scheme 32). Kinetic studies revealed that *β*-H elimination after acrylate insertion led to ester-terminated chain ends, while competing ethylene insertion resulted in acrylate units located in the main chain. Specifically, the copolymer is generated through several competing pathways. *β*-H elimination induced by coordination of acrylates with Ni catalysts can serve as a chain termination pathway, producing an ester chain-ended copolymer. At the same time, the novel Ni alkyl complexes generated after acrylate insertion can undergo further chain growth through competitive ethylene insertion.

DFT studies have also clarified how catalyst structure dictates regioselectivity and activity toward specific polar monomers. Zhu and co-workers [134] studied the mechanism of copolymerization of ethylene and methyl vinyl sulfone (MVS) by phosphinosulfonate and *α*-diimine Pd catalysts (Scheme 32). DFT calculations showed that MVS preferentially underwent 2,1-si-insertion, as the corresponding transition state exhibited the lowest free energy (9.3 kcal/mol) compared with the other three insertion modes, including 1,2-*re* (9.3 vs. 12.7 kcal/mol), 1,2-*si* (9.3 vs. 11.9 kcal/mol), and 2,1-*re* insertion (9.3 vs. 12.6 kcal/mol). For the phosphinosulfonate Pd catalyst, the insertion barrier of MVS was slightly lower than that of ethylene, generating a copolymer in which ethylene and polar monomers were incorporated in both the main chain and at chain ends. In stark contrast, the *α*-diimine Pd catalyst was inactive for the copolymerization of ethylene with MVS. This was attributed to the larger buried volume of the metal center in the *α*-diimine Pd catalyst (% V_bur_ = 73.9) compared to that in the phosphinosulfonate Pd catalyst (% V_bur_ = 68.3), which favored the formation of a stable five- or four-membered palladacycle intermediate that directly resulted in a high barrier for chain propagation.

## 5. Conclusions and Outlook

In this review, we mainly discussed the recent advances of Pd and Ni catalysts for the direct synthesis of F-PE. As stated above, Pd and Ni catalysts enable the direct coordination–insertion copolymerization of ethylene with various industrial commodity polar monomers and show great potential from fundamental research to practical application. Facile synthetic strategies and modular ligand tuning lay the foundation for large-scale production. Rational design of catalysts, including adjustment of axial steric hindrance, electronic modification of the ligand, and the introduction of secondary coordination sphere interactions, allows fine regulation of the copolymerization process and the resulting polymer microstructure.

Although encouraging advances have been achieved so far, many aspects still need to be considered or improved for practical application. (1) For Pd-based systems, copolymers of ethylene with commercial polar vinyl monomers have been successfully prepared. However, most Pd catalytic systems still suffer from low catalytic activity, relatively poor thermal stability, and the formation of low-MW copolymers, which are obstacles on the road to industrialization. According to recent advances, strategies such as the modification of ligand steric hindrance or the use of polar additives can substantially improve the performance of Pd catalysts. Nevertheless, improving the thermal stability of α-diimine Pd catalysts while maintaining high MW and moderate branching density remains a key challenge. (2) Ni catalysts are expected to be a better alternative for commercial application compared to Pd catalysts due to their higher abundance and lower cost. Although the oxophilicity of Ni is higher than that of Pd and makes it more susceptible to poisoning by polar monomers, fine-tuning of the ligand structure can effectively adjust the electronic properties of Ni and counteract the oxophilic effect. Future efforts should focus on designing ligands that simultaneously shield the axial sites and reduce the electrophilicity of the Ni center. This has been exemplified by the shielded phosphinophenolate and dinaphthobarrelene frameworks reported in recent studies. (3) The range of polar monomers catalyzed by Ni catalysts has been expanded from initially limited types to the current commercially used polar vinyl monomers, which is a firm step forward toward practical industrial progress. However, the low MW of the resulting copolymers and the limited monomer incorporation rate remain challenging fundamental problems. Achieving over 10 mol% incorporation of polar monomers while maintaining MW above 10^5^ g/mol is an important target for the next generation of Ni catalysts. Recent breakthroughs with sulfonate-modified [N,O] and bulky [P,O] systems have demonstrated the feasibility of this goal. (4) A full understanding of the mechanism of each catalytic process is essential for the rational design of catalyst structures. Catalyst deactivation in Pd- and Ni-catalyzed copolymerizations of ethylene with polar monomers arises predominantly from two pathways: direct poisoning of the metal center by the heteroatom-containing functional groups, and the formation of stable chelates through intramolecular coordination of the polar functional group to the metal center immediately after monomer insertion. These processes often induce further β-X elimination reactions and represent the main focal points for new catalyst design. In the near future, combining kinetic studies with operando spectroscopy and DFT calculations will be crucial to elucidate the elementary steps of chain propagation, chain transfer, and catalyst dormancy. This is especially important for emerging multinuclear and heterobimetallic systems, where the synergism is not yet fully understood.

In summary, the development of Pd and Ni catalysts for the preparation of F-PE copolymers is rapidly advanced in recent years, but it is still far from industrial practice, and more efforts are needed to improve the balance of high-performance catalysts with economic catalytic efficiency, adjustable polar monomer incorporation rate, controllable MW, and molecular topology, to meet the growing demand for F-PE materials.

## Figures and Tables

**Table 1:** Abbreviations of polar monomer names.

Full Name	Abbreviation	Full Name	Abbreviation
6-chloro-1-hexene	6-Cl-Hex	methyl undec-10-enoate	MU
acrylic acid	AA	methyl vinyl acetate	MVA
allyl acetate	AAc	norbornene	NB
allyl chloride	AC	p-phenylcinnamic acid	p-COOHSt
allyl ethyl ether	AEE	phenyl vinyl sulfone	PVS
acrylonitrile	AN	*tert*-butyl acrylate	*t*BA
butyl vinyl ether	BVE	undec-10-enoic acid	UA
dimethyl itaconate	DMI	but-3-enoic acid (vinyl acetic acid)	VA
5-hexene-1-yl-acetate	HAc	ethenyl acetate (vinyl acetate)	VAc
methyl acrylate	MA	ethenyltrimethoxysilane	VTMoS
methyl methacrylate	MMA	norbornene derivatives	x-NB-x

Note: common names shown in parentheses are provided for easy reference.

## Data Availability

No new data were created or analyzed in this study.
